# Abnormal Biomarkers of Homocysteine Metabolism in Neonates with Conotruncal Heart Defects

**DOI:** 10.1155/2017/7404397

**Published:** 2017-07-27

**Authors:** Piotr Surmiak, Małgorzata Baumert, Magdalena Paprotny

**Affiliations:** Department of Neonatology, School of Medicine in Katowice, Medical University of Silesia, Katowice, Poland

## Abstract

**Objectives:**

The etiology of conotruncal heart defects (CHD) remains unknown; however relation between homocysteine, folate levels, and congenital heart disease was found. With this perspective in mind, the aim of the study was to investigate biomarkers of homosyteine metabolism pathway in mothers and their neonates with CHD.

**Material and Methods:**

Forty-three pairs of mothers and their neonates with CHD and forty pairs of mothers and neonates with nonconotruncal heart defects (non-CHD) were enrolled. The control group (CG) consisted of fifty-nine pairs of mothers and their healthy neonates. For estimating the plasma total homocysteine (tHcy), serum folates, and cobalamin levels, mothers' venous blood samples and umbilical cord blood were taken in all groups.

**Results:**

We observed higher tHcy levels in newborns with CHD in comparison to their mothers and to neonates with non-CHD. Cobalamin levels were significantly lower in neonates with CHD compared to other children. Folates and cobalamin levels were lower in CHD mothers compared to their children.

**Conclusions:**

Elevated homocysteine levels in neonates with CHD and folate metabolism disturbances in their mothers were noticed. The observed differences in homocysteine and cobalamin levels between neonates with CHD suggest the influence of various agents disturbing homocysteine metabolic pathways.

## 1. Introduction

The most common problem of contemporary perinatology is a high number of congenital anomalies of which conotruncal heart defects (CHD) are one of the common defects in the developmental period [1, 2]. Conotruncal heart defects represent an anatomically heterogeneous group of cardiac malformations affecting the outflow tract of the ventricles and the arterial pole of the heart. According to current knowledge, more than 80% of congenital defects with recognized etiology are stimulated by genetic and environmental factors [2]. Chief among the aforementioned influences was a deficiency of essential microelements and vitamins in the preconception period, such as the B-vitamins (i.e., folic acid and cobalamin). Some reports suggested that folates play a key role in utilization reactions of many biogenic compounds, including homocysteine [3, 4].

Recent studies suggested that maternal hyperhomocysteinemia is an independent risk factor for congenital heart defects, by interfering with the development of conotruncal septum of the heart [5, 6].

The aim of our study was to evaluate the differences in total homocysteine (tHcy), folate, and cobalamin levels of maternal and umbilical cord blood samples in groups of children with congenital heart defects.

## 2. Material and Method

A prospective, case-control study was conducted in the Department of Neonatology at the Medical University of Silesia in Katowice between January 2012 and January 2015. The study was approved by the Ethics Committee of the Medical University of Silesia (nr KNW/0022/KB1/25/13).

Among 1,271 childbirths in our unit at the time of the study we enrolled 83 neonates (6.5%) with prenatally diagnosed congenital heart defects.

The control group (CG) is comprised of 59 pairs of healthy mothers residing in the unit during the study and their healthy, full-term newborns.

All mothers from the study group underwent at least two prenatal examinations in which the congenital heart defects were diagnosed. Congenital heart defects in neonates were confirmed by echocardiography performed as soon as possible after delivery.

The exclusion criteria comprised neonates with chromosomal aberrations, complex congenital malformations, newborns from multiple pregnancies, and neonates with evidence of congenital infections, as well as those that were born to mothers with clinical chorioamnionitis. We also excluded mothers who administered medications during pregnancy, which could possibly affect homocysteine and vitamin B metabolism (i.e., folate antagonists, antiepileptic drugs, oral contraceptives, barbiturates, and levodopa) within the period of six months before conception, as well as pregnant women suffering from hypertension, thromboembolic diseases, kidney, and heart defects. Neonates from the control group underwent a cranial ultrasound and an echocardiographic examination after delivery.

All included mothers and their children with prenatally diagnosed congenital heart defect were divided into two subgroups: with conotruncal heart defects (CHD, *n* = 43) and nonconotruncal heart defects (non-CHD, *n* = 40). CHD group included neonates with diagnosed Persistent Truncus Arteriosus, PTA (*n* = 13, 30.1%), Tetralogy of Fallot, TOF (*n* = 11, 25.6%), Interrupted Aortic Arch, IAA (*n* = 6, 13.9%), and Double Outlet Right Ventricle, DORV (*n* = 5, 11.6%).

Nonconotruncal heart defects (non-CHD) comprised children with Atrial Septum Defect, ASD (*n* = 16, 40.0%), Ventricular Septum Defect, VSD (*n* = 15, 37.5%), and Atrioventricular Septal Defect, AVSC (*n* = 9, 22.5%). All congenital anomalies were reported to the Polish Registry of Congenital Malformations.

### 2.1. Laboratory Performances

During childbirth, 5 milliliters of maternal blood was collected from ulnar vein and 5 ml of blood from umbilical artery from the placental side.

All blood samples were collected in EDTA-containing tubes, centrifuged for 10 minutes (2500 rotations/min) and stored at −70°C until full analysis had been made. The following was assessed: folate, cobalamin, and total homocysteine concentrations. Folate and cobalamin in serum were determined with the aid of microparticle enzyme immunoassay (MEIA), using ABBOTT reagent sets in an immunochemical analyzer (AxSYM). Total homocysteine concentration in plasma was determined by an immunochemical method with the fluorescence polarization immunoassay (FPIA) using an IMx analyzer and special ABBOTT sets. All procedures were recommended by the ABBOTT company which produces testing sets used in our examination.

### 2.2. Statistical Analysis

Results were analyzed statistically with the certified program STATISTICA 10 (StatSoft Polska Inc.). The distribution of the data was analyzed by the Shapiro-Wilk test. Results were presented as means and standard deviations or as percentiles of the total. Baseline characteristics and biomarkers of homocysteine metabolism between all study groups were compared using Kruskal-Wallis test, *U* Mann–Whitney test, or chi square tests.

The association between variables was measured by a Spearman's rank correlation test. For all the statistical procedures *p* value < 0.05 was considered to be significant.

## 3. Results

Mothers and their neonates from all investigated groups were comparable to controls with respect to demographic-perinatal characteristics, presented in Table 1.

We have noticed that daily dietary supplementation of folic acid (0.4 mg) was taken by 86.0% (*n* = 37) of mothers from CHD group, 87.5% (*n* = 35) of mothers from non-CHD group, and 89.8% (*n* = 53) from controls. However, those results were not statistically significant (*p* = 0.3). We observed no relevant differences in creatinine levels in all investigated mothers (CHD 0.75 mg/dl, non-CHD 0.72 mg/dl, CG 0.74 mg/dl, *p* = 0.7).

### 3.1. Total Homocysteine Levels

In our study, we observed significantly higher tHcy levels in umbilical cord blood in newborns with CHD compared to their mothers. Significant differences in tHcy concentrations were observed in umbilical cord blood between CHD compared to non-CHD groups. We also noticed higher umbilical cord tHcy levels in CHD neonates compared to controls.

However, we observed no significant differences in tHcy levels in neonates with non-CHD in comparison to their mothers and to controls.

We found no relevant differences in tHcy between mothers in all investigated groups.

**Figure 1:**
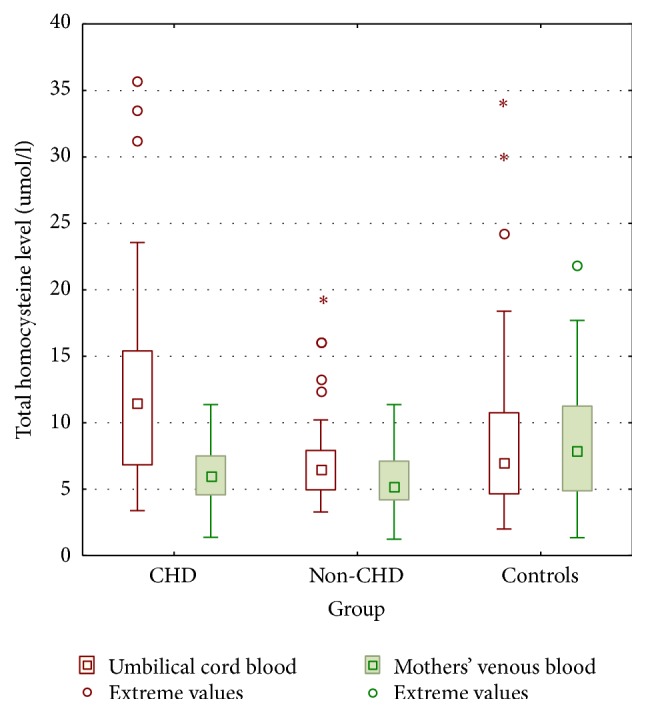
Maternal and umbilical cord blood total homocysteine concentrations in conotruncal heart defects (CHD), nonconotruncal heart defects (non-CHD), and controls (CG). Results presented as means and standard deviations as well as 95% confidence intervals and extreme values. ^*∗*^Extreme values.

All results are presented on Figure 1 and Table 2.

### 3.2. Folate Levels

**Figure 2:**
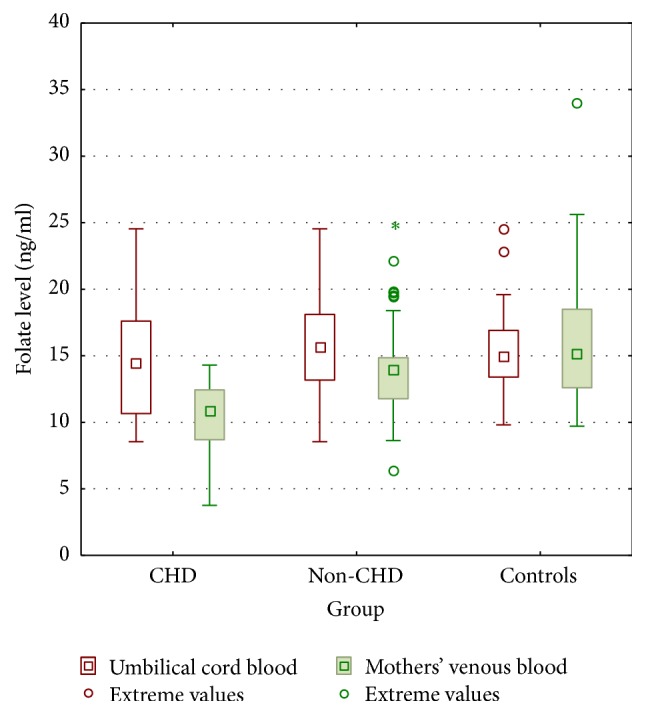
Maternal and umbilical cord blood folate concentrations in conotruncal heart defects (CHD), nonconotruncal heart defects (non-CHD), and controls (CG). Results presented as means and standard deviations as well as 95% confidence intervals and extreme values. ^*∗*^Extreme values.

No differences were shown in umbilical cord folate levels in all investigated children. However, we have noticed significant decrease folate levels in CHD mothers in comparison to mothers from non-CHD and control groups, shown in Figure 2 and in Table 2.

Neonates born with CHD presented significantly higher folate concentrations in comparison to their mothers. However, there were no noticeable differences in folate levels between neonates with non-CHD and their mothers. Similarly, folates levels were comparable in control neonates compared to their mothers.

### 3.3. Cobalamin Levels

**Figure 3:**
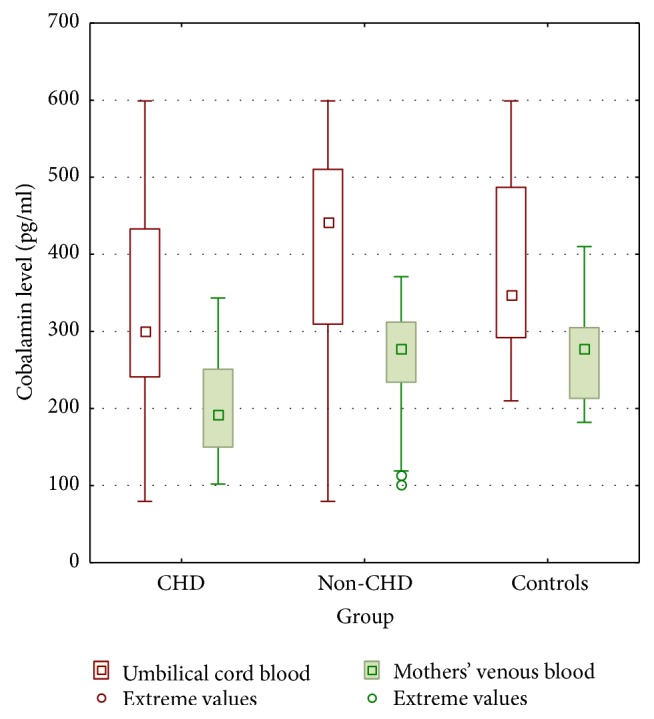
Maternal and umbilical cord blood cobalamin concentrations in conotruncal heart defects (CHD), nonconotruncal heart defects (non-CHD), and controls (CG). Results presented as means and standard deviations as well as 95% confidence intervals and extreme values.

Cobalamin levels were significantly lower in umbilical cord blood in CHD group compared to others, presented on Figure 3 and Table 2.

We have noticed relevant lower cobalamin levels in mothers from CHD and non-CHD group compared to their children. However, similar results were observed in control mothers in comparison to their neonates. We observed significant differences in cobalamin levels in CHD mothers in comparison to mothers from non-CHD and control groups.

### 3.4. Correlations

We observed correlation between tHcy and folate levels (*r* = −0.64, *p* = 0.002), as well as tHcy and cobalamin levels (*r* = −0.31, *p* = 0.04) in CHD group.

We also observed correlation between folate levels in all investigated mothers and umbilical cord tHcy levels (*r* = −0.63, *p* = 0.03).

## 4. Discussion

In this study, we analyzed biomarkers of the folate-dependent homocysteine pathway metabolism in neonates with congenital heart defects and their mothers. We noticed elevated homocysteine levels in umbilical cord blood in neonates born with conotruncal heart defects in comparison to newborns with nonconotruncal heart defects. However, some studies indicate maternal elevated homocysteine level as a main risk factor for congenital heart defects in their offspring [7, 8]. We did not observe such differences in homocysteine levels between investigated mothers. Perhaps those differences are the result of the proportionally small group of participants in our study. Additionally, our study demonstrated decreased cobalamin levels in neonates with congenital heart defects and their mothers compared to controls. Some studies presented cobalamin and folate deficiency in pregnancy complicated by congenital anomaly, which may suggest inadequate daily folic acid and cobalamin dietary supplementation during pregnancy [9, 10]. Different results are presented by Hobbs et al., where vitamin B-12 and folic acid concentrations did not differ significantly between mothers with congenital heart defect and control subjects [8]. However, other authors suggested that congenital heart anomalies are associated with low maternal folate as well as with hyperhomocysteinemia [11, 12]. Authors revealed that cobalamin and folate administration may help to reduce the development of cardiac malformations [13, 14].

The etiology of conotruncal heart diseases is complex, with both environmental and genetic causes. It has been well documented that hyperhomocysteinemia, which is often accompanied by the defects of folic acid metabolism, is associated with the occurrence of congenital defects, and it seems to be an independent risk factor of conotruncal heart defects [15–17].

Based on our results, we postulated that mothers' homocysteine levels had no direct influence on the development of conotruncal heart defects. Thus, we suggested that hyperhomocysteinemia and decreased folate levels observed in umbilical cord blood may be associated with the disturbances in homocysteine pathway metabolism in newborns with CHD. Zhao et al. found in children with CHD a gene mutation coding for an enzyme, which plays an important role in the homocysteine remethylation process [18]. It is probable that, in fetuses with CHD, the excess homocysteine is metabolized by remethylation, as we discovered a decreased concentration of folic acid in children with CHD compared to those with non-CHD.

According to Solanky et al., remethylation of Hcy to methionine using methyl donation from folate is the prevalent pathway in the human placenta, indicating a marked reliance on folate availability [19]. Consequently, vitamin B deficiency in mothers whose offspring have congenital malformations additionally causes a disturbance in the main pathway of Hcy remethylation in the placenta and results in an increased transfer of Hcy from maternal to fetal circulation.

The basis for the observed abnormal metabolic profile among neonates with conotruncal heart defects and their mothers cannot be defined without further analysis of relevant genetic and environmental factors. Therefore, confirmation by future prospective multicentre cohorts is needed.

## 5. Conclusions

Elevated homocysteine levels in neonates with conotruncal heart defects and folate metabolism disturbances in their mothers were noticed.

The observed differences in homocysteine and cobalamin levels between neonates with congenital heart defects suggest the influence of various agents disturbing homocysteine metabolic pathways.

## Figures and Tables

**Table 1:** Demographic characteristics of investigated neonates and their mothers.

Variable	Conotruncal heart defects (*n* = 43)	Nonconotruncal heart defects (*n* = 40)	Controls (*n* = 59)	*p* value
Mothers				
(i) Age (years)	26 [4.3]	29 [5.8]	31 [5.4]	0.5
(ii) Primigravida [*n*, %]	26, 60.5%	27, 67.5%	35, 59.3%	0.4
(iii) Dietary supplementation of folic acid [*n*, %]	37, 86.0%	35, 87.5%	53, 89.8%	0.3
(iv) Delivery mode: caesarean section [*n*, %]	26, 60.5%	22, 55.0%	30, 50.8%	0.1

Newborns				
(i) Gender female [*n*, %]	20, 46.5%	23, 57.5%	30, 50.8%	0.7
(ii) Gestational age (weeks)	38.5 [3.6]	37 [2.7]	39.5 [4.2]	0.6
(iii) Birth weight (g)	3090 [620]	3313 [840]	3230 [750]	0.4
(iv) Head circumference (cm)	34.5 [2.0]	34.5 [1.5]	34.0 [2.5]	0.1
(v) Body length (cm)	53.0 [1.5]	54.5 [2.5]	53.5 [1.5]	0.5
(vi) Apgar 1st min [*n*, %]				
0–3 pts	2, 4.7%	1, 2.3%	0	0.6
4–7 pts	5, 11.6%	8, 20.0%	8, 13.6%	
8–10 pts	36, 83.7%	32, 80.0%	51, 86.4%	
(vii) Apgar 5th min [*n*, %]				
0–3 pts	0	0	0	0.5
4–7 pts	2, 4.7%	6, 15.0%	5, 8.5%	
8–10 pts	41, 95.3%	34, 85.0%	54, 81.5%	

Results are shown as mean and standard deviation [SD] or percentile; *p* value from Kruskal-Wallis test or chi square test.

**Table 2:** Total homocysteine (tHcy), folate, and cobalamin levels in umbilical cord blood and mothers' venous blood samples in all investigated groups.

Variable	Conotruncal heart defects (*n* = 43)	Nonconotruncal heart defects (*n* = 40)	Controls (*n* = 59)	*p* value
Umbilical cord blood				
(i) tHcy level [*μ*mol/l]	12.6 [2.4]	7.7 [2.3]	8.1 [2.6]	0.01
(ii) Folate level [ng/ml]	13.7 [4.1]	14.3 [2.7]	15.0 [1.3]	0.2
(iii) Cobalamin level [pg/ml]	300.5 [95.7]	425.1 [120.7]	325.4 [105.2]	<0.01

Mothers				
(i) tHcy level [*μ*mol/l]	7.2 [2.0]	7.1 [2.4]	6.8 [3.3]	0.3
(ii) Folate level [ng/ml]	10.8 [2.2]	14.1 [1.8]	15.5 [3.5]	<0.01
(iii) Cobalamin level [pg/ml]	198.8 [98.5]	269.8 [60.9]	258.7 [101.1]	<0.01

Results are presented as mean and standard deviation [SD]; *p* value from Kruskal-Wallis test.
